# Zoledronic acid inhibits macrophage/microglia-assisted breast cancer cell invasion

**DOI:** 10.18632/oncotarget.1201

**Published:** 2013-08-19

**Authors:** Eva Rietkötter, Kerstin Menck, Annalen Bleckmann, Katja Farhat, Meike Schaffrinski, Matthias Schulz, Uwe-Karsten Hanisch, Claudia Binder, Tobias Pukrop

**Affiliations:** ^1^ Department of Hematology/Oncology, University Medical Center, 37099 Göttingen, Germany; ^2^ Department of Medical Statistics, University Medical Center, 37099 Göttingen, Germany; ^3^ Department of Cardiovascular Physiology, University Medical Center, 37099 Göttingen, Germany; ^4^ Institute of Neuropathology, University Medical Center, 37099 Göttingen, Germany

**Keywords:** Zoledronic acid, macrophages, microglia, metastasis, tumor microenvironment

## Abstract

The bisphosphonate zoledronic acid (ZA) significantly reduces complications of bone metastasis by inhibiting resident macrophages, the osteoclasts. Recent clinical trials indicate additional anti-metastatic effects of ZA outside the bone. However, which step of metastasis is influenced and whether this is due to direct toxicity on cancer cells or inhibition of the tumor promoting microenvironment, is unknown. In particular, tumor-associated and resident macrophages support each step of organ metastasis and could be a crucial target of ZA.

Thus, we comparatively investigate the ZA effects on: i) different types of macrophages, ii) on breast cancer cells but also iii) on macrophage-induced invasion. We demonstrate that ZA concentrations reflecting the plasma level affected viability of human macrophages, murine bone marrow-derived macrophages as well as their resident brain equivalents, the microglia, while it did not influence the tested cancer cells. However, the effects on the macrophages subsequently reduced the macrophage/microglia-induced invasiveness of the cancer cells.

In line with this, manipulation of microglia by ZA in organotypic brain slice cocultures reduced the tissue invasion by carcinoma cells. The characterization of human macrophages after ZA treatment revealed a phenotype/response shift, in particular after external stimulation.

In conclusion, we show that therapeutic concentrations of ZA affect all types of macrophages but not the cancer cells. Thus, anti-metastatic effects of ZA are predominantly caused by modulating the microenvironment. Most importantly, our findings demonstrate that ZA reduced microglia-assisted invasion of cancer cells to the brain tissue, indicating a potential therapeutic role in the prevention of cerebral metastasis.

## INTRODUCTION

Zoledronic acid (ZA) demonstrated in pre-clinical studies as well as clinical trials anti-metastatic activity during different steps of tumor progression. Several clinical trials analyzed the effects of ZA on overall survival of breast cancer patients. Both, the ABCSG-12 trial as well as the ZO-FAST trial revealed an increased disease-free survival of patients receiving an adjuvant therapy in combination with ZA [1, 2]. In the AZURE trial a tendency to an increased overall-survival of patients treated with an adjuvant therapy in combination with ZA, compared to the adjuvant therapy alone, was identified [2]. Interestingly, the analysis of a subgroup of patients receiving neo-adjuvant chemotherapy together with ZA demonstrated a significant decrease in the primary tumor mass compared to the neo-adjuvant chemotherapy alone [3].

Using different mouse models of mammary tumors, it has been shown that ZA reduces the burden of bone but also lung and liver metastases, it diminishes the amount of macrophages in the primary tumor (tumor-associated macrophages) and the vascularization. In these studies ZA treatment increased not only tumor-free but also overall survival [4, 5]. Translational studies in patients with solid tumors revealed a continued decrease in VEGF serum levels after treatment with ZA [6-8]. Additionally, formation of new blood vessels has been shown to be inhibited by several bisphosphonates [9]. Furthermore, for breast cancer patients immunomodulating properties of ZA could be validated [10]. For example, a modulation of γδ-T-cell functions with eventually tumor-inhibiting effects has been documented for bisphosphonates [11].

Regardless ZA influences different aspects of the tumor microenvironment the majority of report explains the results of the clinical trials by direct toxic effects on the breast cancer cells. This is based on different *in vitro* observations. ZA has been shown to inhibit proliferation, migration, invasion to increase apoptosis and decrease adhesion to the bone of malignant cells [12-14]. Here it must be remembered, that mostly these effects are achieved at high ZA concentrations.

Nevertheless, the most frequently investigated and confirmed mechanism of action of ZA is mediated by the inhibition of osteoclasts, the resident macrophages of the bone. This leads to decreased bone resorption and diminished osteoclast-derived growth factors, as the tumor progression factor TGF-β [15, 16].

Despite the inhibitory effects of ZA on osteoclasts, the impact on other macrophage populations outside the bone has been barely investigated. A possible explanation for the scant attention of other macrophage populations is the low concentration of ZA outside the bone. However, since *in vivo* studies already indicated an influence of ZA on the tumor-associated macrophages (TAM) in the primary tumor mass, we were interested in whether ZA would affect other types of macrophages. For this purpose, we not only focus on the primary tumor and TAM, which effectively support the first steps of metastasis, but also investigate the last step of metastasis, the colonization of distant organs. Our recent results already revealed that high concentrations of the bisphosphonate clodronate influences microglia, the resident macrophage-like cells of the central nervous system (CNS). Microglia assist the colonization of the brain by breast cancer cells. Clodronate reduced the microglia-induced invasion and interfered with the active transport of carcinoma cells by microglia [17, 18]. Moreover, it reduced the microglia response against intruding benign epithelial cells and the activated damage response caused by the destruction of the carcinoma cells.

However, clodronate belongs to the non-nitrogen-containing bisphosphonates, while ZA is a nitrogen-containing bisphosphonates, showing two different modes of action. Clodronate is metabolized into a toxic ATP analogue which impairs mitochondrial function and eventually induces apoptosis [19]. In contrast to that, nitrogen-containing bisphosphonates interfere with the mevalonate pathway and hence inhibit prenylation, an essential process for the activation of small GTPases [20, 21]. Furthermore, nitrogen-containing bisphosphonates increase the accumulation of the ATP analogue ApppI and, therefore, induce apoptosis [22].

Considering that nitrogen-containing bisphosphonates are taken up by endocytosis and the fact that macrophage-like cells exhibit a much higher endocytotic activity than tumor cells, it is reasonable that some of the systemic anti-tumor effects of ZA observed in the clinical trials are mediated by impeding the tumor supporting activities of different macrophage-like populations also in low concentrations. Thus, we systematically analyzed the effects of ZA on terminally differentiated human macrophages from the peripheral blood, murine bone marrow-derived macrophages (BMDM) and resident macrophage-like cells of the CNS, the microglia, in comparison to its impact on breast cancer cells. Moreover, we investigated the effects of ZA on the tumor-promoting interaction between cancer cells and macrophages by measuring its influence on human macrophage- and microglia-induced invasion using Boyden chamber assays as well as an organotypic brain slice cancer cell coculture. Most important all experiments were performed with therapeutic-relevant concentrations of ZA.

## RESULTS

### ZA is more cytotoxic for macrophages than for human breast cancer cells

**Figure 1:**
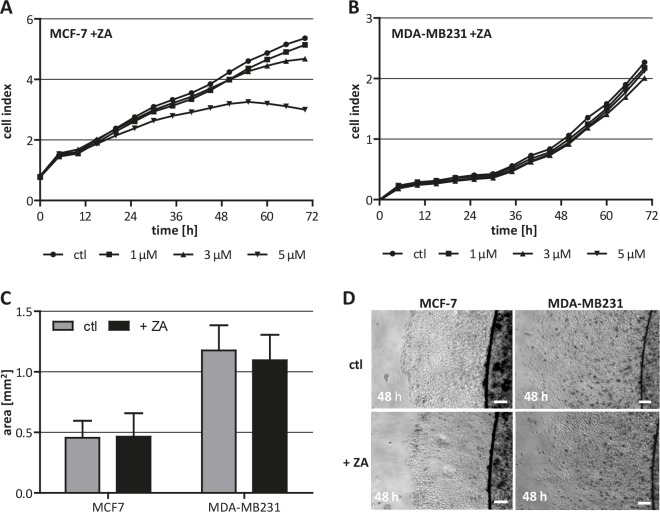
Cytotoxicity of ZA on breast cancer cells (A+B) High concentrations of ZA affect viability of MCF-7 but not of MDA-MB231. MCF-7 (A) and MDA-MB231 (B) were treated with either 0 μM (circle), 1 μM (square), 3 μM (triangle) or 5 μM (inverse triangle) ZA. Cell proliferation was measured over 72 h using the xCELLigence system and is indicated as cell index. (C+D) Migration capacity of MCF-7 and MDA-MB231 is not affected by ZA. ECM-based migration assays for MCF-7 and MDA-MB231 over 48 h in the absence (gray bars, top pictures) and presence of 2 μM ZA (black bars, bottom pictures). Scale bars indicate 200 μm.

To be able to discriminate direct effects of ZA on breast cancer cells from impacts on different macrophage populations, we first analyzed the toxicity of this drug for two human breast cancer cell lines. MCF-7 (luminal A subtype) and MDA-MB231 (basal-like subtype) cells [23, 24] were treated with increasing concentrations of ZA while recording their cell index (proliferation) using the xCELLigence system. MCF-7 cells showed an only moderately reduced cell index when treated with the highest concentration of ZA (5 μM), while the drug did not affect the proliferation of MDA-MB231 at any concentration tested (Fig. 1 A, B). An important characteristic of metastasizing tumor cells is their migratory activity. To further clarify if this capacity is affected by ZA we performed extra-cellular matrix (ECM)-based migration assays and measured the area which the tumor cells covered within 48 h. The results revealed no change in the migration capacity of neither MCF-7 nor MDA-MB231 by treatment with 2 μM ZA (Fig. 1 C-D).

**Figure 2:**
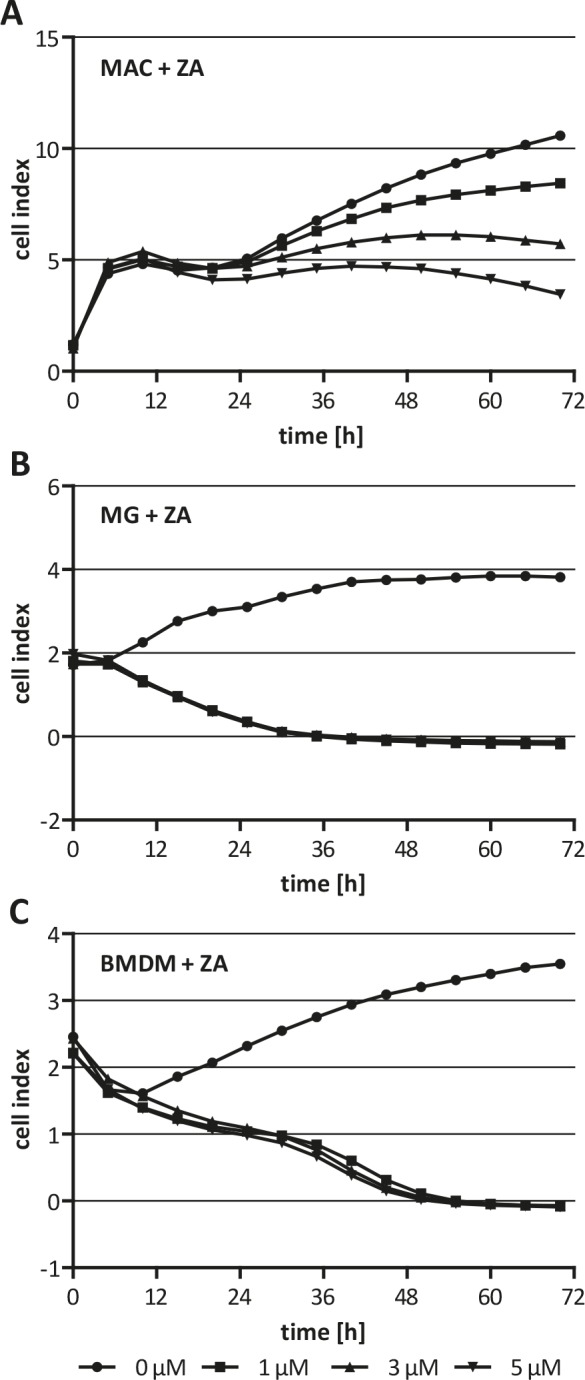
Cytotoxicity of ZA on macrophages (A) Human macrophages (MAC), (B) microglia (MG) and (C) BMDM were treated with either 0 μM (circle), 1 μM (square), 3 μM (triangle) or 5 μM (inverse triangle) ZA. Cell proliferation/morphology was measured over 72 h using the xCELLigence system and is indicated as cell index. While viability of MAC is affected dose-dependently by ZA, viability of MG and BMDM is affected at the lowest concentration tested after 36 h and 48 h treatment, respectively.

Since ZA cannot pass the plasma membrane passively but is internalized by endocytosis we hypothesized that macrophages are more sensitive to ZA. To prove this we treated three different macrophage populations with increasing concentration of ZA. The xCELLigence measurements revealed that all three populations were sensitive to ZA already at the lowest concentration tested. ZA decreased the cell index for human macrophages in a dose dependent manner (Fig. 2 A). Notably, murine microglia and BMDM were already completely inhibited by ZA at the lowest concentration. (1 μM) after 36 h and 48 h of treatment, respectively (Fig 2 B, C). This is in great contrast to the results obtained with the cancer cells supporting our hypothesis that the anti-tumor effects of ZA could be mediated by an inhibition of different macrophage-like populations.

### ZA decreases human macrophage-induced invasiveness of MCF-7 cells

**Figure 3:**
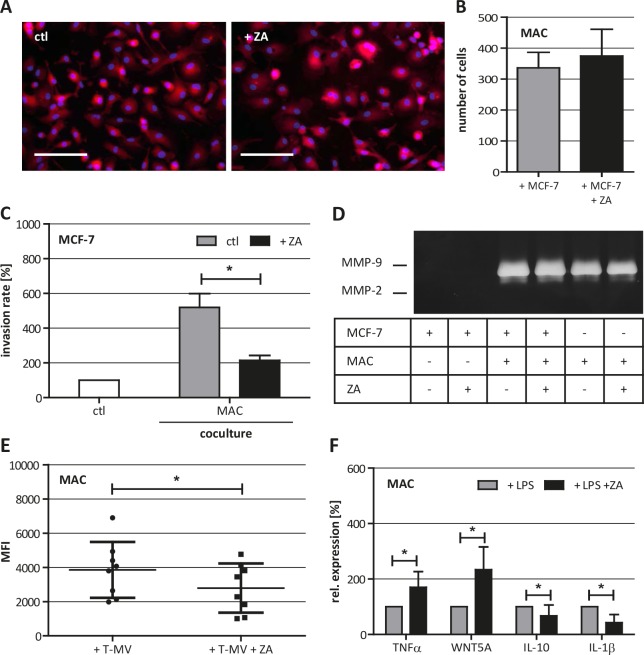
Characterization of ZA-treated human macrophages (MAC) (A) Phalloidin-TRITC staining of untreated MAC (left picture) and MAC treated with 1 μM ZA (right picture) showing the same cell morphology under both conditions. Scale bars indicate 100 μm. (B) Migration assay for MAC in coculture with MCF-7 in the absence (gray bar) and presence of 1 μM ZA (black bar) reveal no difference in the number of migrated cells (means ± SD, n = 8). (C) ZA decreases the MAC-induced invasiveness of MCF-7 cells shown by microinvasion assay of MCF-7 cells alone (white bar) and in coculture with MAC in the absence (gray bar) or presence of 1 μM ZA (black bar). Invasiveness is indicated as percentage of the control MCF-7 cells alone (means ± SD, n = 3, *P < 0.01). (D) Gelatine zymography of cell culture supernatants from MCF-7 cells alone, + ZA, + MAC, + MAC and ZA, MAC alone and MAC +ZA reveal no difference in the secretion of MMP-9 after ZA treatment. (E) ZA decreases T-MV uptake in MAC shown by FACS analysis. Cells were treated with 1 μM ZA (square) or diluent (circle) for 48 h before adding 5 μg PKH26-labeled T-MV for another 24 h. T-MV uptake is indicated as mean fluorescent intensity (MFI) of MAC (means ± SD, n = 7, *P < 0.05). (F) ZA alters LPS-induced expression of *TNFα*, *WNT5A*, *IL-10* and *IL-1β* measured by qRT-PCR in MAC treated with 100 μg/ml LPS alone (gray bars) or 100 μg/ml LPS + 1 μM ZA (black bars). Relative expression levels are indicated as percentage of the condition MAC + 100 μg/ml LPS (means ± SD, n ≥ 4, *P ≤ 0.05).

One μM ZA already decreased the cell index of human macrophages. This led to the question whether the morphology of human macrophages is influenced by ZA. Therefore, we stained human macrophages with PKH26 and analyzed their cell shape by fluorescence microscopy. Obviously, the morphology of human macorphages was not affected by ZA (Fig. 3 A). Next, we wanted to know if the migratory capacity of human macrophages is altered by ZA and performed a coculture-based migration assay. Human macrophages were cocultivated with MCF-7 cells alone or in the presence of 1 μM ZA. The number of migrated cells was determined after 11 h of coculture. As shown in Fig. 3 B, ZA did not affect the migratory capacity of the macrophages.

Previously, we have shown that human macrophages enhance the invasiveness of weakly invasive MCF-7 breast cancer cells [25, 26]. Moreover, we demonstrated that microglia-induced invasion is antagonized by the bisphosphonate clodronate [18]. Thus, we analyzed the effect of ZA on human macrophage-induced invasiveness of MCF-7 cells in a modified Boyden chamber. The invasion rate of MCF-7 was measured after 96 h of coculture with human macrophages in the presence of 1 μM ZA, a concentration having no effects on the malignant cells as shown above. The results demonstrated a significant decrease in the human macrophage-induced invasiveness of MCF-7 cells (Fig. 3 C).

### ZA does not influence matrix metalloprotease (MMP) secretion but decreases microvesicle (MV)-uptake and alters LPS-induced gene expression in human macrophages

The capability of a cell to degrade ECM is an important determinant for its tissue invasion and is mediated, amongst others, by MMPs. Since it has already been shown that ZA can decrease the secretion of MMPs [14] we were interested in whether this could explain the effect of ZA on macrophage-induced invasiveness of malignant cells. By performing gelatin zymography we could show that MCF-7 cells alone did not secrete detectable amounts of MMP-2 and MMP-9. In contrast to that, we could confirm previous results that MMP-9 is secreted by human macrophages and that the secretion is increased under coculture conditions with MCF-7 cells [25, 26]. However, the secretion of MMP-9 was not affected by 2 μM ZA (Fig. 3 D).

Unpublished data of our group show that the human macrophage-induced invasiveness of malignant cells, is partly mediated by the release of tumor microvesicles (T-MV) which are subsequently ingested by human macrophages. Microvesicles are small extracellular vesicles (diameter 100-1000 nm) which could be released from the cellular plasma membrane of virtually all cells [27]. Uptake of T-MV by human macrophages can be mediated, amongst other mechanisms, by phagocytosis and is therefore dependent on small G-proteins. Thus, we were interested in whether ZA has an impact on the uptake of T-MV by macrophages. By incubating human macrophages with fluorescence-labeled T-MV derived from the breast cancer cell line MCF-7 we could show that the uptake of T-MV is significantly decreased by 1 μM ZA (Fig. 3 E).

Next, we analyzed if ZA, beside its impact on the uptake of T-MV, also changes the expression profile of human macrophages induced by external stimulus. Human macrophages were treated with 100 μg/ml LPS alone or in combination with 1 μM ZA before quantifying the expression of *TNFα*, *WNT5A*, *IL-10* and *IL-1β* by qRT-PCR. All genes showed an altered expression in the presence of ZA, as compared to the sole treatment with LPS. The expression of *TNFα* was enhanced, whereas expression of *IL-1β* was significantly decreased by ZA (Fig. 3 F). Taken together, while there is no obvious effect on human macrophage morphology, migratory capacity as well as MMP production, ZA seems to interfere with endocytosis of T-MV and the response to a pathogenic stimulus, such as LPS. Most importantly, these findings clearly demonstrate that low concentrations of ZA could repolarize TAM in the primary tumor mass without affecting the carcinoma cells.

### Microglia-induced invasion of MCF-7 is decreased by ZA

**Figure 4:**
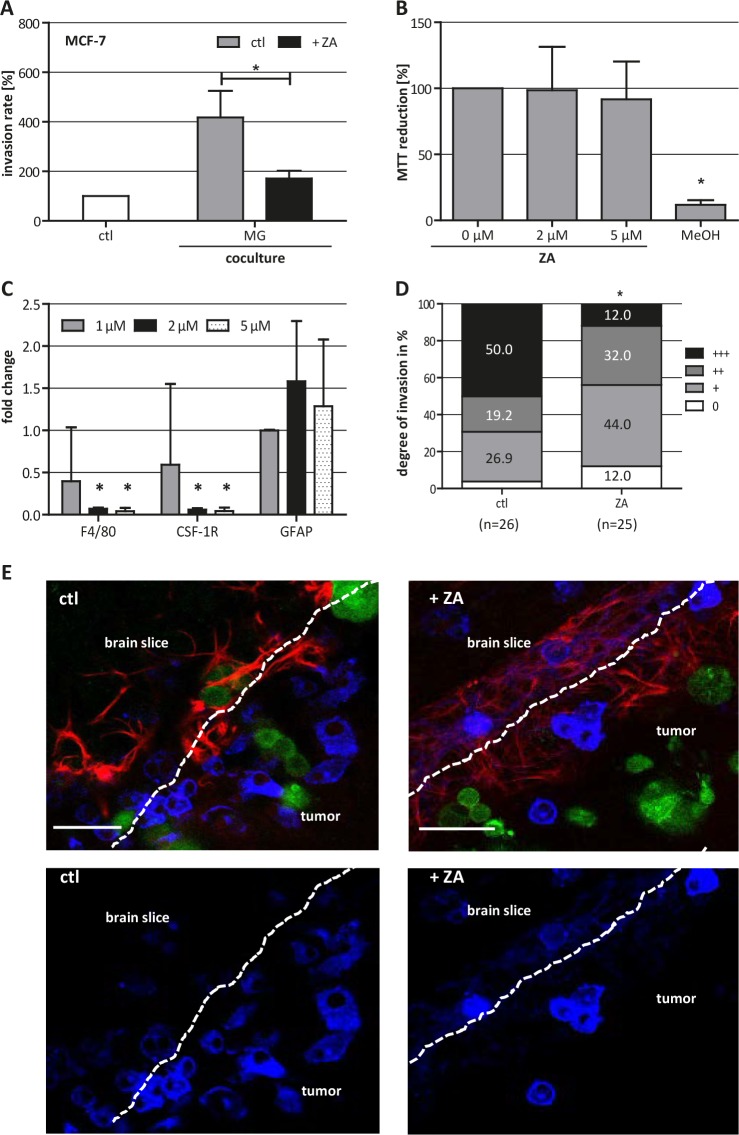
ZA decreases microglia (MG)-induced invasiveness of MCF-7 cells (A) ZA decreases MG-induced invasiveness of MCF-7 cells shown by microinvasion assay of MCF-7 cells alone (white bar) and in coculture with MG in the absence (gray bar) or presence of 1 μM ZA (black bar). Invasiveness is indicated as percentage of the control MCF-7 cells alone (means ± SD, n = 3, *P < 0.05). (B) MTT assay of organotypic brain slices treated with ZA at the concentrations indicated or methanol (MeOH) reveal no toxic effects of ZA on the tissue. MTT reduction is given as percentage of untreated slices (means ± SD, n = 6, *P < 0.001). (C) ZA decreases the expression of *F4/80* and *Csf-1R* but not *Gfap* in organotypic brain slices measured by qRT-PCR. Tissue was treated with 1 μM (gray bars), 2 μM (black bars) or 5 μM (black doted bars) ZA for 72 h. Relative expression levels are indicated as fold changes to the untreated control (means ± SD, n = 3, *P < 0.001). (D) ZA decreases MCF-7 cell invasion in organotypic whole-brain slice cocultures. Tumor cell invasion was quantified in the absence (left bar) and presence (right bar) of 2 μM ZA (n = number of slices; +++ = highliy invasive; ++ = moderately invasive; + = low invasive; 0 = non-invasive) (*P = 0.01). (E) Picture of organotypic brain slices cocultured with GFP-labeled MCF-7 cells (green) and stained with isolectin B4-674 (blue) and αGFAP-TRITC (red) in the absence (left) and presence (right) of 2 μM ZA. Scale bars indicate 50 μm.

To demonstrate that ZA could also affect the resident macrophage-like populations in target organs of metastasis other than the bone, we investigated the effects of ZA on different aspects during cerebral metastasis. In modified Boyden chamber assays, conducting indirect coculture of breast cancer cells and microglia, ZA reduced the microglia-induced invasion of MCF-7 cells nearly to the basal level (Fig. 4 A). Most importantly, these effects were detected at concentrations (1 μM) which showed no influence on the carcinoma cells.

To confirm these results, we used our recently established brain slice coculture model. First, we measured the viability of the brain tissue upon treatment with increasing concentrations of ZA for 72 h by a MTT conversion assay. The MTT measurements revealed no change in brain slice viability and, therefore, excluded any direct toxic effect of ZA on the living tissue (Fig. 4 B). Nevertheless, 2 μM and 5 μM ZA significantly decreased the expression of the microglia markers *F4/80* and *Csf-1r*, whereas it did not affect the expression of the astrocyte-specific marker *Gfap*, pointing to a specific decrease of microglia in the tissue (Fig. 4 C).

Next, we quantified the invasion of MCF-7 cells into living brain tissue in untreated slices and those treated with 2 μM ZA. Treatment with 2 μM ZA significantly reduced the invasion of MCF-7 into the living brain slice, in particular, the rate of high invasion (+++) was lowered. In the control condition (ctl) 50% of all slices revealed high invasion of the breast carcinoma cells while this was decreased to only 12% in the ZA treatment group (Fig. 4 D). Once more, the concentration of ZA used in this assay demonstrated no effect on MCF-7 cells in terms of viability and migration. Interestingly, confocal microscopy revealed that there was no complete depletion of microglia adjacent to the 3D tumor plug in the brain tissue after treatment with ZA. This is unexpected based on the significant reduction of two microglia marker shown by qRT-PCR. Moreover, microglia were still able to enter the tumor plug in the control group as well as in the ZA treatment group. For this reason, the effects of ZA treatment apparently are not mediated by the depletion of microglia but more by influencing their tumor-promoting capacity.

## DISCUSSION

To date ZA and other bisphosphonates undoubtedly influence the course of osteolytic bone lesions not only of breast cancer patients. This is due to the enrichment of ZA in the bone tissue where very high concentrations of ZA subsequently inhibit the resident macrophages, the osteoclasts. Despite this well-known effect on osteoclasts, a systematical analysis for other types of macrophages or macrophage-like cells does not exist.

However, *in vivo* experiments and most important the results of clinical trials in breast cancer point to additional anti-tumor effects in the primary tumor mass as well as other metastatic sites than bone, where bisphosphonates are present in much lower concentrations. In addition, the authors mainly explain these anti-tumor effects by direct toxicity on the carcinoma cells and less on the macrophages in the primary tumor or the resident ones at the metastatic sites.

However, our results demonstrate that already plasma concentrations of ZA affect all kinds of tested macrophages as well as microglia, most likely because their tremendous rate of phagocytosis. Furthermore, the macrophage/microglia-induced invasion of the breast cancer cells was reduced by these low concentrations without affecting the carcinoma cells itself. Our results for human macrophages revealed that obviously, this effect is not due to direct dramatic changes in the morphology, migration or MMP release. In contrast, ZA treatment slightly influences the response to secondary stimuli and seems to shift the physiological response of human macrophages. Since TAM are an important source of VEGF and involved in tumor angiogenesis the phenotype shift of macrophages could explain the translational findings in the above mentioned studies. Previous results already demonstrated that a phenotype shift of murine macrophages reduced the tumor progression [28] and also microglia-induced invasion was inhibited by shifting in an inflammatory phenotype [18]. Therefore, ZA seems to manipulate the education or persistence of the tumor-promoting macrophage phenotype. At least, the experiment analyzing the T-MV uptake by human macrophages indicates a possible disruption of the paracrine communication between carcinoma cells and human macrophages. In general, extracellular vesicles are involved in the formation of the metastatic niche [29]. Furthermore, it has been demonstrated for MV to foster tumor progression as well as transfer different derivatives of cancer cells (e.g. RNA, proteins) to adjacent cells [30, 31]. Thus, manipulation of this communication axis between tumor cells and tumor-promoting macrophages in the primary tumor, as well as resident macrophages in the metastatic target organs, seems a very promising therapeutic strategy in treatment or even prevention of distant metastasis. Our results of the brain slice coculture model support this assumption. This model already enabled us to describe an unknown mechanism of carcinoma cell invasion, the microglia-assisted invasion [18]. There carcinoma cells misuse a physiological damage response and repair program of the resident cells to invade the brain. Furthermore, we identified the microglia as the main mediator of the tumor promoting effects [17]. Here we demonstrate that ZA reduced breast carcinoma invasion to brain tissue by selective manipulation of the microglia. Thus, ZA seems a very promising drug not only to reduce skeletal complications but also to prevent metastasis to other organs with resident macrophage populations (e.g. liver, lung and brain). The clinical trials already support this assumption. However, future studies have to show the effects on prevention of cerebral metastasis. At the present time point no suitable *in vivo* models for cerebral metastasis exist to test this hypothesis. A spontaneous breast cancer model or syngeneic injection model with measurable secondary metastasis to the brain is still not available. Thus, retrospective analysis of previous or future clinical trials could be a more appropriate and valid option.

Nevertheless, knowing that the incidence of cerebral metastasis is increasing and ZA revealed significant effects on the brain tissue colonization, improving the chemical structure or the delivery of ZA to the brain should be taken into account. For example liposomal packed bisphosphonates, as used in many *in vivo* studies, could be a very promising way to increase their concentration and anti-tumor effects, at least outside the bone. In particular, the passage of the blood-brain barrier could be improved.

In conclusion ZA inhibits various tumor-promoting macrophages during all steps of metastasis, in the primary organ and at the metastatic site. It seems that this is more due to a phenotype shift of the macrophages than simply toxic effects. In particular, it could influence the communication between macrophages and cancer cells which could be very important in the formation of the metastatic niche. Thus, improving organ delivery/ distribution of ZA could be an important task for the future and subsequently increase their anti-metastatic effects in other organs with more impact on overall survival. In general this effect could eventually expand to other tumor types where TAM also influences the prognosis.

## Material and Methods

### Cells and media

If not indicated otherwise, substances were purchased from Sigma (Munich, Germany). The human breast cancer cell lines MCF-7 and MDA-MB231 were obtained from the American Type Culture Collection (Rockville, USA). The cell lines were cultivated in RPMI-1640 medium (PAA, Cölbe, Germany) supplemented with 10% heat inactivated fetal bovine serum (FCS; Sigma, Munich, Germany).

### Isolation and differentiation of human macrophages

Human macrophages were derived from peripheral blood mononuclear cells according to the double density technique established previously [32]. Briefly, after centrifugation over a Ficoll-Isopaque density gradient (Biochrome, Berlin, Germany) mononuclear cells were subsequently purified by an iso-osmotic Percoll gradient (GE, Freiburg, Germany). Monocytes were differentiated into macrophages over 7 days of culture in Teflon-coated cell culture bags (Cellgenix, Freiburg, Germany) in the presence of 2 ng/ml rhM-CSF (Immunotools, Friesoythe, Germany).

### Isolation of BMDM

Murine BMDM were isolated as described previously [33], with slight modifications. Briefly, femurs of 8-12 week old NMRI mice were flushed with growth medium (DMEM (Biochrome, Berlin, Germany) + 10% heat inactivated FCS (Sigma, Munich, Germany), 5% heat inactivated NHS (Gibco, Darmstadt, Germany), 30% L929 conditioned medium, 2 mM L-glutamine, 0,01 mM sodium pyruvate, 0,05 mM 2-mercaptoethanol, 100 U/ml penicillin and 100 mg/ml streptomycin) and cultivated overnight in cell culture dishes (Nunc, Wiesbaden, Germany) to remove fibroblasts. Non-adherent cells were collected and cultured for 6 days in non-coated culture dishes (Sarstedt, Nümbrecht, Germany) in growth medium. For experiments BMDM were cultured in DMEM + 10% heat inactivated FCS + 15% L929 conditioned medium.

L929 conditioned medium, as a source of M-CSF, was prepared as previously described [34].

### Isolation of murine microglia

Primary microglia cell cultures from newborn (P0) NMRI mice were prepared and cultured as previously described [35]. After 10-14 days, microglial cells were plated in cell culture plates or inserts and used 24 h later for experiments.

### Metabolism assay

Cell metabolism was analysed by measurement of MTT (2,3-diphenyl-5-methyltetrazolium chloride; Sigma, Munich, Germany) conversion according to standard procedures. Organotypic brain slices were treated with ZA for 72 h before measuring MTT reduction.

### Proliferation assay

Cell proliferation assays were performed using the xCELLigence RTCA DP system (Roche, Mannheim, Germany). A cell density of 1 × 10^3^ (MDA-MB231), 4 × 10^4^ (MCF-7, BMDM) or 8 × 10^4^ (microglia, human macrophages) cells per well were plated and proliferation/morphology was analyzed for 72 h in quadruplets.

### Microinvasion assay

Invasion was measured using an artificial basement membrane in a modified Boyden chamber, where the cellular components were grown without direct cell-to-cell contact as described previously [25]. Briefly, the membrane consisted of a polycarbonate (10 μm pore diameter; Nucleopore, Pleasanton, USA) and was coated with Matrigel (ECM gel; R&D Systems, Wiesbaden, Germany) diluted 1/3 in serum-free RPMI 1640 media. 1 × 10^5^ MCF-7 cells were seeded into the upper well of the chamber, the lower well was filled with medium. For coculture experiments 2 × 10^5^ human macrophages RPMI + 1% FCS (DMEM + 10% FCS for microglia and BMDM) were seeded in transwell inserts (Nunc, Wiesbaden, Germany). The transwells were inserted into the upper well of the Boyden chamber and 1 μM zolendric acid was added. After 96 h the floating and adherent cells in the lower well were removed and counted. All experiments were performed at least in triplicate.

### Coculture-based migration assay

For the coculture-based migration assay 0.75 × 10^5^ human macrophages were seeded in RPMI-1640 + 1% FCS medium into inserts (BD Biosciences, Heidelberg, Germany) containing 3 μm pores. Before placing inserts into 24-wells containing 1 × 10^5^ MCF-7 cells, both human macrophages and MCF-7 cells were pre-treated with 1 μM ZA for 2 h. After 11 h of coculture in the presence of 1 μM ZA, inserts were placed into medium with 5 mM calcein (Sigma-Aldrich, Munich, Germany) for 1 h to stain vital human macrophages. Finally, cells that had not migrated were removed by scraping from the upper side of the insert, while migrated cells on the bottom side were analyzed by fluorescence microscopy using the Axiovert 200M microscope (Zeiss, Göttingen, Germany).

### Organotypic slice coculture model

Organotypic brain slice coculture experiments were performed as previously described [18]. Briefly, NMRI mice (P7-P10) were decapitated. Brains were removed under aseptic conditions and placed in ice-cold MEM medium (Invitrogen, Darmstadt, Germany) containing 0.2 mM glutamine, 100 U/ml penicillin, 100 mg/ml streptomycin (Sigma, Munich, Germany) and 4.5 mg/ml glucose (Braun, Melsungen, Germany). The forebrain was dissected from the brainstem and attached onto an aluminium block by cyanoacrylate glue (Renfert, Hilzingen, Germany). Horizontal whole brain sections of 350 μm were obtained with a vibratome (Leica VT1200S; Leica, Wetzlar, Germany). Brain slices were transferred onto a 0.4 mm polycarbonate membrane in a transwell tissue insert (Falcon, model 3090, BD, Heidelberg, Germany), which was inserted into a 6-well dish. Slices were incubated in 50% MEM, 25% Hanks' balanced salt solution (Gibco, Darmstadt, Germany), 25% normal horse serum, 0.2 mM glutamine, 100 U/ml penicillin, 100 mg/ml streptomycin and 4.5 mg/ml glucose. After 24 h 1 × 10^5^ tumor cells, suspended in 15 μl RPMI with 75% extracellular matrix gel (R&D Systems, Wiesbaden, Germany), were placed next to the intact outside of the slice using a spacer with a diameter of 5 mm. The spacer was removed after 6 h, and the tumor cells were allowed to invade for 96 h. To quantify the invasion rate of tumor cells we used a scoring system from 0 − +++ (0 = no invasion, +++ = strong invasion). Zolendric acid (2 μM) was applied at 0 h, 24 h and 48 h. Fluorescence staining of the coculture was performed as previously described [17]. The method is also illustrated in a scientific video [36].

### RNA isolation

RNA from tissue was isolated with a modified Trizol (Invitrogen, Darmstadt, Germany) method incorporating a DNaseI (Roche, Mannheim, Germany) digestion step. RNA from cells was isolated using the “High Pure RNA isolation kit” (Roche, Mannheim, Germany). Reverse transcription was performed with the iScript Master Mix (BioRad, Munich, Germany).

### qRT-PCR

Quantitative RT-PCR was performed as previously described [18]. The following mRNA specific, intron-spanning primers were used: mmF4/80, mmCsf-1r, mmGfap, hsTNFα, hsIL-10, hsIL-1β and hsWNT5A (sequences see supplementary table S1). All qRT-PCRs were performed using the HT 7900 system (Applied Biosystems, Darmstadt, Germany). Gene expression was analysed by using the SDS Software Version 2.4 (Applied Biosystems) normalizing the expression to two housekeeping genes, mmTbp/mmGapdh and hsHPRT1/ hsGNB2L1.

### ECM-based migration assay

Migration assays were performed as previously described [18]. Migration was analyzed by measuring distances after 48 h using the Axiovert 200M microscope and the Axiovision Rel.4.6.3 Software (Zeiss, Göttingen, Germany). Zoledronic acid was added at 0 h at a concentration of 2 μM.

### Zymography

Zymography was performed as previously described [25]. Briefly, cell supernatants (5 μl) and lysates (10 μg) were separated on 8% SDS-polyacrylamide gels containing 1 mg/ml gelatine. After incubating gels in renaturation buffer for 24 h they were stained with Coomassie brilliant blue.

### Isolation of T-MV

For the isolation of T-MV, MCF-7 cells were cultured for 48 h in RPMI-1640 medium containing particle-free, heat-inactivated FCS. The particle-free FCS was generated through pelleting endogenous MV and exosomes by ultracentrifugation at 100,000 g overnight. Cell culture supernatants were consecutively centrifuged at 750 g for 5 min and at 1,500 g for 15 min to remove cells and debris. This was followed by ultracentrifugation for 45 min at 14,000 g and 4°C to precipitate the MV. The pellet was washed once in PBS and resuspended in 200μl PBS for protein quantification using the Lowry method (D_c_ protein assay, Bio-Rad, Munich, Germany).

### MV-uptake experiments

T-MV were stained with the red-fluorescent membrane dye PKH26 (Sigma-Aldrich, Munich, Germany) according to the manufacturer's instructions. Human macrophages were pre-incubated with ZA (1 μM) for 48 h and then stimulated with PKH26-labeled T-MV (5 μg/ml) for 24 h. Uptake was analyzed measuring the fluorescence intensity of the cells using the FACSCanto II flow cytometer (BD, Heidelberg, Germany).

### Statistics

Using the Student's t-test and the Kolmogorov-Smirnov test the significance of the differences between groups was tested.

## Supplementary Table S1


