# Supplementary material for: Zoledronic acid inhibits macrophage/microglia-assisted breast cancer cell invasion
Source: Oncotarget. 2013 Aug 19;4(9):1449–60. doi: 10.18632/oncotarget.1201 (PMC3824537; doi:10.18632/oncotarget.1201)
Supplement: Supplementary file 1 [file oncotarget-04-1449-s001.docx]

Supplementary Table S1: Primer Sequences

| Gene | fwd | rev |
| --- | --- | --- |
| hsGNB2L1 | AACCCTATCATCGTCTCCT | CAATGTGGTTGGTCTTCAG |
| hsHPRT1 | TATGCTGAGGATTTGGAAAGG | CATCTCCTTCATCACATCTCG |
| hsIL-10 | CTTGCTGGAGGACTTTAAGG | CTGGATCATCTCAGACAAGG |
| hsIL-1β | TACGATCACTGAACTGCAC | CAAAGGACATGGAGAACAC |
| hsTNFα | TCTCTAATCAGCCCTCTGG | CTACAACATGGGCTACAGG |
| hsWNT5A | AGGGCTCCTACGAGAGTGCT | GACACCCCATGGCACTTG |
| mmCsf-1r | CACCATCCACTTGTATGTC | CTCAACCACTGTCACCTC |
| mmF4/80 | TGATTCAGACGGAGTACCT | CAGCCAACATTCATCTTGTC |
| mmGapdh | CATCTTGGGCTACACTGAG | CTGTAGCCGTATTCATTGTC |
| mmGfap | AACCTGGCTGCGTATAGAC | CCAGCGATTCAACCTTTCTC |
| mmTbp | TTCCCAGCTAAGTTCTTAGAC | CCAGGAAATAATTCTGGCTC |
